# Effects of chronic exposure to biomass pollutants on cardiorespiratory responses and the occurrence of exercise‐induced bronchoconstriction in healthy men

**DOI:** 10.14814/phy2.70368

**Published:** 2025-05-09

**Authors:** Pierre Lofuta Olenga Vuvu, Malgorzata Klass, Nathalie Pauwen, Augustin Mboko Kipula, Philippe van de Borne, Alain van Muylem, Silvia Perez‐Bogerd, Gael Deboeck

**Affiliations:** ^1^ Cardiopulmonary Rehabilitation Unit, Physical Medicine and Rehabilitation Department, University Clinics of Kinshasa University of Kinshasa Kinshasa Democratic Republic of the Congo; ^2^ Research Unit in Rehabilitation Sciences, Faculty of Human Movement Sciences Université Libre de Bruxelles Brussels Belgium; ^3^ Research Unit in Biometry and Exercise Nutrition, Faculty of Human Movement Sciences Université Libre de Bruxelles Brussels Belgium; ^4^ Cardiopulmonary Exercise Laboratory, Faculty of Human Movement Sciences Université Libre de Bruxelles Brussels Belgium; ^5^ Cardiology Department, Erasme University Hospital Université Libre de Bruxelles Brussels Belgium; ^6^ Pulmonology Department, Erasme University Hospital Université Libre de Bruxelles Brussels Belgium; ^7^ Epidemiology and Biostatistics Unit, Public Health School Université Libre de Bruxelles Brussels Belgium

**Keywords:** 15 m − ISWRT, aerobic capacity, biomass air pollution, cardiovascular responses, Charcoal, exercise‐induced bronchoconstriction

## Abstract

Exposure to charcoal biomass (CB) pollutants affects the cardiorespiratory system. We assessed cardiopulmonary responses (CPR) to exercise in charcoal producers (CPs) compared to farmers and evaluated the prevalence of exercise‐induced bronchoconstriction (EIB). Forty‐five CPs and 36 farmers, healthy males aged 23–39, completed a 15‐m Incremental Shuttle Walk and Run Test (15‐m ISWRT). Air quality index (AQI) and CO intoxication were measured, CPR was assessed through heart rate (HR), blood pressures (SBP, DBP), and spirometry at rest, peak exercise, and during recovery at 5 and 15 min. Aerobic capacity (VO_2_ max) was estimated from the distance covered during the 15‐m ISWRT, and EIB was defined as a >10% decrease in FEV1 from baseline values. AQI was worse in charcoal workplaces, and CPs had higher CO intoxication than farmers (*p* < 0.0001). Both groups reached maximal exercise %HRmax: 84 (82–89) versus 84 (80–89), *p* = 0.37 and showed similar predicted VO_2_ max 36.2 (31.1–43.1) versus 38.9 (32.2–43.7) mL/kg/min, *p* = 0.60. However, after ISWRT, CPs had lower FEV1 than farmers (2.9 ± 0.6 vs. 3.3 ± 0.6 L, *p* < 0.003) and slower recovery. EIB prevalence was higher in CPs (60.0% vs. 27.8%, *p* = 0.006). Chronic exposure to CB increases EIB in healthy CPs, suggesting heightened airway hyperreactivity.

## 1 INTRODUCTION

Exposure to biomass pollutants significantly affects the cardiorespiratory system at rest and contributes to chronic conditions such as chronic obstructive pulmonary disease (COPD) and arterial hypertension (Landrigan et al., 2018). Charcoal production, a widespread activity in low‐income countries, represents a major source of biomass pollution (Idowu et al., 2023; Olujimi et al., 2016). Charcoal production involves high‐temperature pyrolysis, releasing toxic smoke and dust, including soot, polycyclic aromatic hydrocarbons, volatile organic compounds, ultrafine particles, and micro and macro‐particles (Lofuta Olenga Vuvu et al., 2024; Olujimi et al., 2016). Charcoal producers (CPs) engaged in this activity for several years face daily physically demanding tasks such as cutting wood, maintaining kilns in activity, packaging or handling charcoal sacks, increasing ventilation rates, and chronic exposure to biomass pollutants (Idowu et al., 2023; Lofuta Olenga Vuvu et al., 2024).

Acute exposure to biomass impairs exercise capacity and cardiovascular responses to exercise, with mixed effects on pulmonary function in healthy subjects and in certain workers (Ferguson et al., 2017; Swiston et al., 2008; Unosson et al., 2013). However, the cardiopulmonary effects of chronic exposure to biomass, particularly from charcoal, remain poorly documented (Koehle, 2024; You et al., 2022). Exposure to air pollutants negatively affects respiratory adaptations to exercise and increases the risk of exercise‐induced bronchoconstriction (EIB) (Levai et al., 2016; Price, Sewry, et al., 2022). EIB, defined by a reduction of more than 10% in forced expiratory volume in the first second (FEV1) within 15 min post‐exercise (Greiwe et al., 2020), is prevalent in 15% of healthy adults and exceeds 30% in hyperactive subjects exposed to air pollutants (Price, Sewry, et al., 2022; Reinhard‐Groebli & Nicod, 2017). The impact of chronic exposure to biomass pollution on EIB and exercise capacity in African adults has not been investigated (Agodokpessi et al., 2012; Idowu et al., 2023).

EIB and exercise capacity are commonly assessed using spirometry after maximal incremental exercise tests in laboratories or field‐based walking and running tests (Holland et al., 2014; Mikawa & Senjyu, 2011; Rundell & Sue‐Chu, 2010). The Incremental Shuttle Walk and Run Test (ISWRT) is a validated and reliable field‐based alternative replicating the laboratory test (Holland et al., 2014; Mikawa & Senjyu, 2011). It challenges the cardiorespiratory system to maximal intensity, estimating maximal exercise capacity (VO_2_ max) and facilitating EIB detection (Léger & Lambert, 1982; Mikawa & Senjyu, 2011; Singh et al., 1994).

Given that CPs face chronic exposure to occupational charcoal biomass pollution and perform intense PA in these polluted conditions (Idowu et al., 2023; Lofuta Olenga Vuvu et al., 2024), we hypothesized that CPs would exhibit impaired cardiopulmonary responses to exercise and a higher risk of developing EIB during incremental exercise testing compared to a control group living in the same environment but not exposed to charcoal biomass pollution.

## 2 METHODS

### 2.1 Design and population

This randomized analytical study was carried out from June to August 2021 in rural areas of Kinshasa and Central Kongo provinces, Democratic Republic of Congo (DRC). The sample size was calculated using G*Power (version 3.1.9.7) (Faul et al., 2007), estimating a 40% prevalence of EIB in the exposed group (Levai et al., 2016; Reinhard‐Groebli & Nicod, 2017), compared to 15% in the control group (Agodokpessi et al., 2012). Considering an alpha risk of 0.05, a power (1‐beta) of 0.8, and an allocation ratio of 0.9, a total of 97 participants (51 CPs and 46 controls) were required. Healthy male participants aged 20–40 years, with seniority of at least 2 years in their occupation, and a resting FEV1 above 80% of the predicted value were included. Exclusion criteria included physical, mental, or sensory disabilities, or known cardiopulmonary, metabolic, or neurological diseases. Ultimately, 45 CPs and 41 farmers living in the same environment (control group) were recruited from a prior study on COPD and hypertension risks in rural workers (Lofuta et al., 2025; Lofuta Olenga Vuvu et al., 2024).

### 2.2 Exercise testing protocol

Although the 20‐m ISWRT is typically recommended for young subjects with a high level of PA such as athletes, we decided to use the 15‐m ISWRT because the 15‐m distance is a valid alternative to the 10‐ or 20‐m ISWRT for assessing aerobic capacity and cardiopulmonary response to intense exercise suitable for healthy young adults or potentially fragile (Mikawa & Senjyu, 2011; Van Hove et al., 2015). The 15‐m ISWRT was conducted on a flat ground, with the course marked every meter and delimited at both ends by cones placed 0.5 meters before the course boundaries, following guidelines (Holland et al., 2014; Mikawa & Senjyu, 2011). Participants' walking speed ranged from 2.7 km/h^−1^ (level 1) to 12.6 km/h^−1^ (level 12), increasing by increments of 15 m/min^−1^ (Mikawa & Senjyu, 2011). An audio recording set the pace of the test and informed participants in advance when transitioning to the next level and when speed increased. The distance covered was recorded in meters, determined by the number of laps (30‐m) completed and the distance covered at the last level. Reasons for stopping the test, along with the perceived exertion assessed using the modified Borg scale, were also recorded (Radtke et al., 2019).

### 2.3 Outcomes

Primary outcomes were FEV1 and estimated VO_2_ max. FEV1 was measured at baseline (after 10 min of seated rest), immediately post‐exercise, and during recovery at 5 and 15 min. EIB was defined as a decrease of more than 10% of FEV1 from baseline at two distinct time points post‐exercise: at the end andduring recovery (Carlsen et al., 2008; Greiwe et al., 2020; Hallstrand et al., 2018). VO_2_ max was estimated using Mikawa's formula: 14.56 + 0.02 × Distance (Mikawa & Senjyu, 2011). Secondary outcomes included heart rate (HR), systolic blood pressure (SBP), and diastolic blood pressure (DBP) measured at rest, immediately post‐exercise, and during recovery at 1, 5, and 15 min. HR values at exercise peak (HRp) were expressed as a percentage of predicted maximal HR (HRmaxpred = 220—age) (Camarda et al., 2008). Maximal exercise test was confirmed if HRp exceeded 80% of HRmaxpred (Parsons et al., 2013; Radtke et al., 2019).

### 2.4 Preparation and environmental monitoring

Exercise testing was conducted outdoors under recommended conditions for EIB assessment, with temperatures maintained between 20 and 25°C, relative humidity ≤40%, and wind speed <9 km/h (Carlsen et al., 2008; Hallstrand et al., 2018; Parsons et al., 2013). To ensure optimal air quality and dry conditions suitable for exercise testing, daily weather forecasts were supplemented with Air Quality Index (AQI) measurements. AQI was monitored using an EG Air® Temtop sensor (M2000, Elitech Technology, Inc., USA). Used in line with the manufacturer's recommendations, it also provided information on pollution levels at participants' workplaces. Participants were instructed to abstain from engaging in intense PA for 48 h prior to the ISWRT. On the day of testing, they were required to wear a T‐shirt, shorts, and sports shoes to ensure comfort and safety during the exercise and were advised to have a light meal at least 1 h before testing. A detailed explanation and demonstration of the ISWRT protocol were provided a day before the test and reiterated immediately prior to its execution to ensure full understanding and compliance.

### 2.5 Data collection

Exercise tests occurred between 8:30 and 11:30 a.m., with all measurements conducted by the same experimenter specialized in cardiopulmonary physiology and rehabilitation. Baseline sociodemographic data (age, seniority, smoking status), PA levels determined using WHO Global Physical Activity Questionnaire (GPAQ) (World Health Organization, 2002), anthropometrics, and body composition measured using (SECA 217® stadiometer and TANITA® TBF300, respectively). Resting cardiovascular parameters: SBP and DBP were measured using a blood pressure monitor (model HEM‐8705‐WM, Omron Relion, Vernon Hills, Illinois, USA), and HR was taken using a chest strap HR monitor (model FT1; Polar Electro Inc., Kempele, Finland). Pollutants exposure of participants was estimated using exhaled air carbon monoxide (EACO) and carboxyhemoglobin (%COHb) levels measured with a calibrated MicroCO Meter® (Micro Medical Ltd., Rochester, UK). Pulmonary function parameters: FEV1 and FVC were measured through Spirometry following ERS and ATS guidelines (Hallstrand et al., 2018; Parsons et al., 2013), using the Spirolab MIR II® spirometer, with predicted values corrected for ethnicity (Stanojevic et al., 2022). The order of passage of the exercise test was random from a list, and participants were anonymized and assigned numeric codes for data analysis to ensure that the statistician was blinded.

### 2.6 Ethics and registration

The study protocol was registered and approved by the Ethics Commission of the DRC Ministry of Health no. 200/CNES/BN/PMMF/2020. Written informed consent was obtained from all participants. The study procedures and data collection were conducted in accordance with the World Medical Association's Declaration of Helsinki.

### 2.7 Statistical analysis

Data collected were analyzed with SPSS 28.0 (IBM corp., NY, USA), and figures were created using GraphPad Prism 8.0.2 software. The distribution of data was checked, and continuous data respecting normal distribution are presented as mean ± standard deviation; if not, as median and interval quartile range (25th–75th). Categorical data are presented as frequency (*n*) and proportion (%).

Comparisons for continuous data between CPs and farmers were conducted using *t*‐tests or Mann–Whitney *U* tests, as appropriate. Outcome comparisons between groups and within each group for the time points (baseline, end of exercise, and all recovery time points) were performed using Friedman's test with Dunn's correction for HR, SBP, DBP, and ANOVA mixed model with Bonferroni correction for FEV1. Delta values (mean) and percent changes of FEV1, FVC, and FEV1/FVC were calculated to assess the differences between after ISWRT and baseline values within each group. Categorical variables, including EIB prevalence, were analyzed using Chi‐square or Fisher's exact tests as appropriate, with a relative risk estimate. The two‐sided statistical significance was set at *p* < 0.05.

## 3 RESULTS

### 3.1 Baseline characteristics of the participants

**FIGURE 1:**
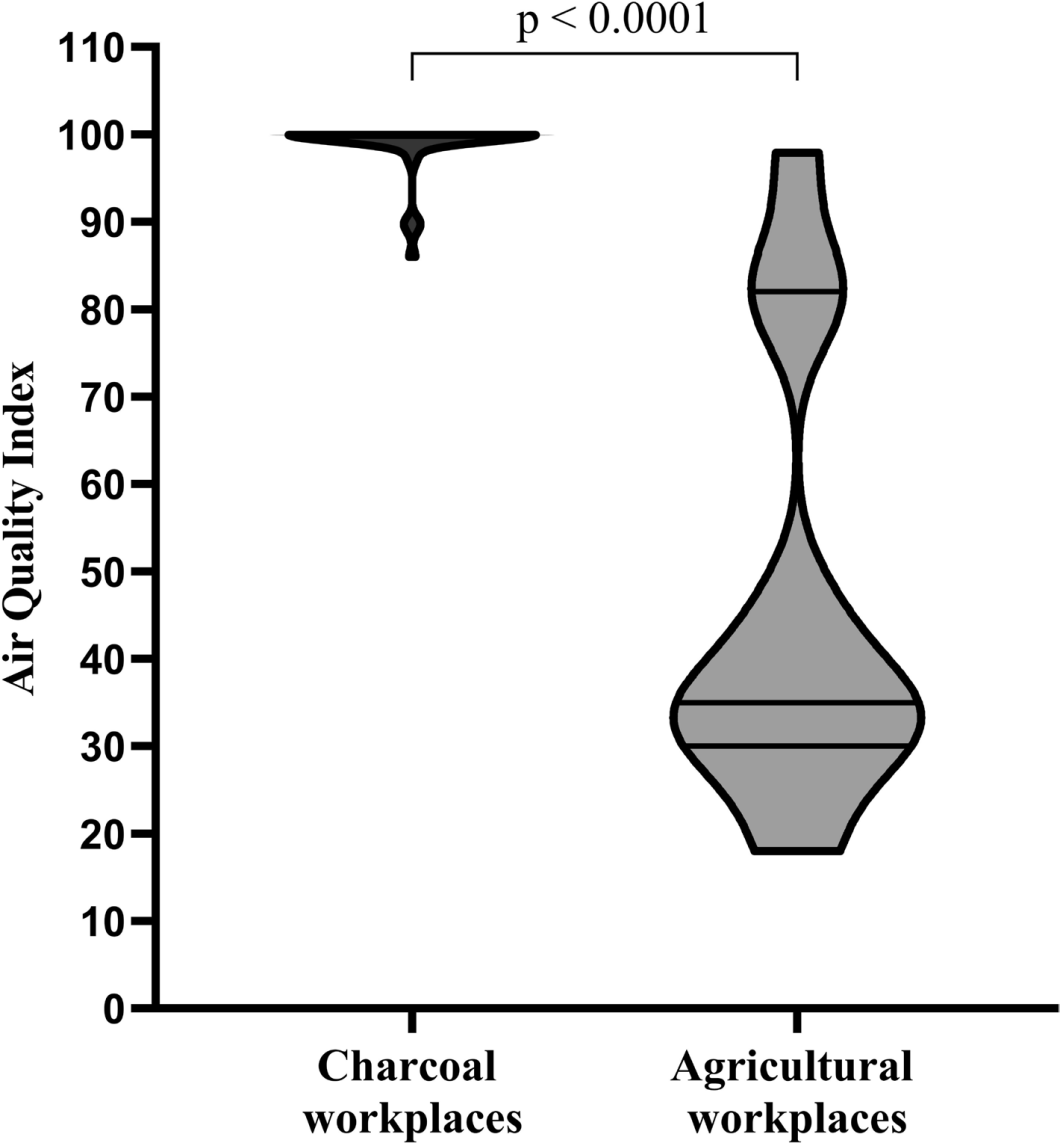
Violin plots of Air quality index in workplaces of participants. Data are expressed as minimum‐to‐maximum ranges. The lines in the violin plots express the medians of the respective data, *p*‐value was calculated using Mann–Whitney *U* test.

**FIGURE 2:**
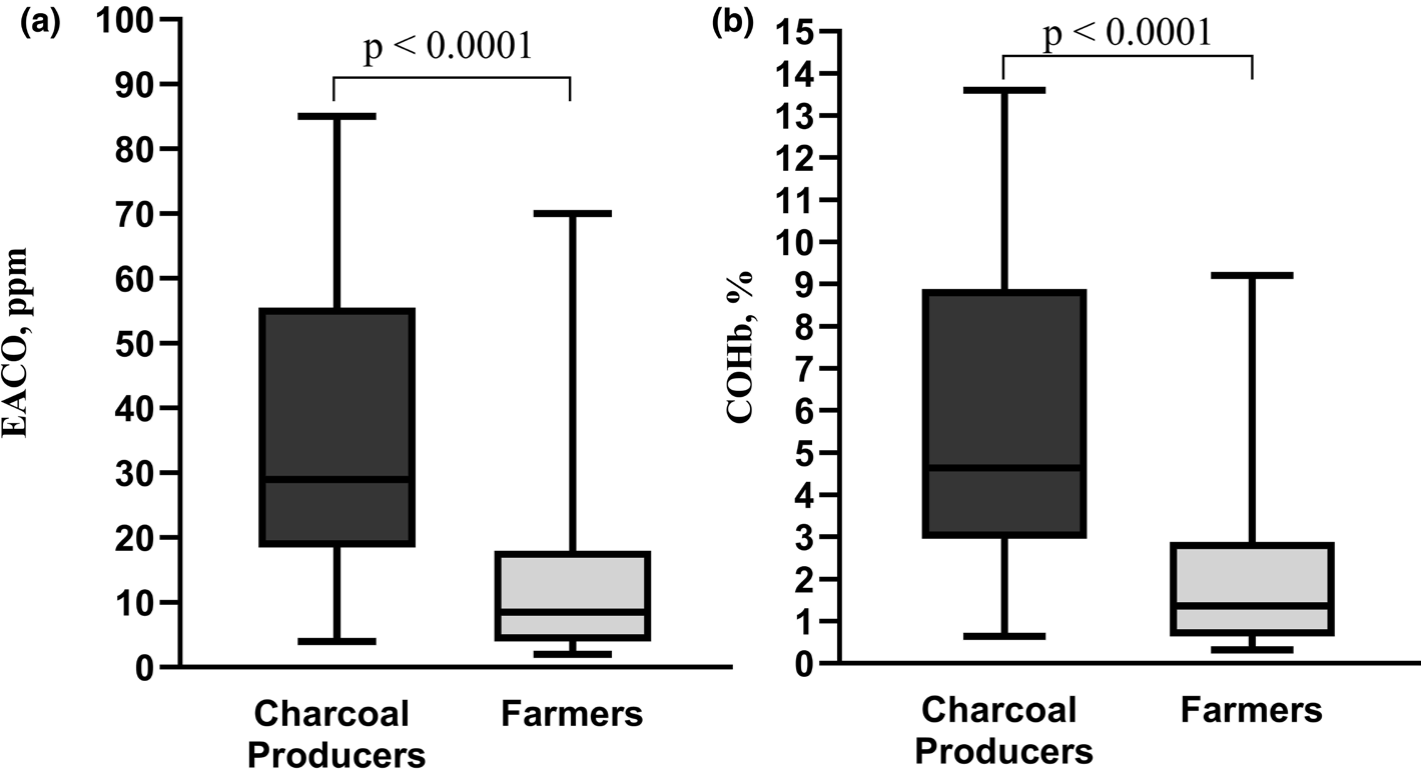
The level of carbon monoxide intoxication in the participant groups. Comparison of Charcoal producers versus Farmers: (a) EACO: exhaled air carbon monoxide; (b) COHb: carboxyhemoglobin. Data were expressed in minimum‐to‐maximum ranges, *p*‐value was calculated using Mann–Whitney *U* test.

Of the 45 CPs and 41 farmers recruited, five farmers were withdrawn from the study because of improper realization of spirometry after exercise. Data from 81 participants, including 45 CPs and 36 farmers, were analyzed, yielding a statistical power of 84%. Charcoal workplaces were significantly more polluted than agricultural workplaces (*p* < 0.0001; Figure 1). Consequently, CPs exhibited markedly higher levels of EACO and COHb compared to farmers (p < 0.0001; Figure 2A,B). Both groups were comparable in age, height, BMI, lean mass, seniority at work, and proportion of smokers. They reported about 5 years of seniority of occupational exposure, with relatively high, but similar participation in moderate and intense PA at work, during leisure time, and active travel (Table 1). Pulmonary function at rest was similar in both groups, except for a lower % predicted of FEV1 in CPs (*p* = 0.02; Table 1).

**TABLE 1:** Baseline characteristics of charcoal producers (exposed group) and farmers (control group).

	Charcoal Producers (*n* = 45)	Farmers (*n* = 36)	*p*‐value
Sociodemographic			
Age, years	32 (25–36)	29 (23–39)	0.29
Seniority at work	5.0 (3.8–9.0)	5.5 (3.0–11.5)	0.85
Height, cm	165 (159–170)	166 (161–173)	0.19
BMI, kg/m^2^	20.4 ± 1.7	21.1 ± 2.2	0.10
Lean Mass, kg	47.5 (44.6–52.1)	50 (45.0–55.3)	0.32
Smoking			
Yes	21 (46.7)	11 (30.6)	0.17
Physical activity level (MET min/week)			
Moderate	6227 ± 1715	5756 ± 2522	0.32
Intense	5760 (1920–9600)	5040 (1800–8160)	0.56
Active travel	1440 (900–2400)	1320 (720–1920)	0.43
Work	11,520 (4920–14,400)	8640 (6480–14,040)	0.42
Global	12,131 ± 4989	11,960 ± 5250	0.88
Pulmonary function at rest			
SpO2, %	98 (97–98)	98 (97–98)	0.73
FEV1, L	3.2 ± 0.6	3.5 ± 0.7	0.33
FEV1, %pred	96 ± 15	104 ± 14	0.02
FVC, L	3.9 (3.5–4.7)	4.2 (3.8–4.8)	0.21
FVC, %pred	106 (93–113)	107 (93–120)	0.38
FEV1/FVC, %	79 ± 12	81 ± 10	0.43

*Note*: Data are presented as median interval quartile (25th–75th), mean ± standard deviation for the continuous data, or frequency (percents) for the categorical data.

Abbreviations: %pred, predicted percentages; FEV1, forced expiratory volume in 1 s; FVC, forced vital capacity; SpO2, partial saturation in oxygen.

### 3.2 Maximal exercise capacity and perceived exertion

Both groups covered the same distance and had similar estimated VO_2_ max. All performed a maximal exercise test (HRp >80% of HRmaxpred) and reported an identical score on the modified Borg scale (Table 2). Reasons for stopping the ISWRT were equally distributed.

**TABLE 2:** Comparisons between charcoal producers and farmers at the end of Incremental Shuttle Walk and Run Test.

	Charcoal producers (*n* = 45)	Farmers (*n* = 36)	RR (CI 95%)	*p*‐value
End of ISWT				
FEV1, L	2.9 ± 0.6	3.3 ± 0.6	—	0.003^a^
FVC, L	3.9 (3.3–4.4)	4.1 (3.5–4.9)	—	0.39^b^
FEV1/FVC, %	74.2 ± 17.2	81.4 ± 20.6	—	0.086^a^
HRp	160 ± 10	159 ± 12	—	0.73^a^
%HRmaxpred	84 (82–89)	84 [80–89]	—	0.37^b^
Covered distance, m	1080 (837–1425)	1200 (874–1303)	—	0.51^b^
Estimated VO_2_ max, mL/kg/min	36.2 (31.1–43.1)	38.9 (32.2–43.7)	—	0.60^b^
Modified Borg score	7 (6–8)	8 (6–9)	—	0.99^b^
EIB				
Yes	27 (60.0)	10 (27.8)	1.8 (1.2–2.7)	0.006^c^
Reasons for stopping ISWT				
Dyspnoea	12 (26.7)	17 (47.2)	—	0.09^d^
Leg tiredness	19 (42.2)	10 (27.8)	—	0.21^d^
Dyspnoea and leg tiredness	9 (20.0)	4 (11.1)	—	0.20^d^

*Note*: Data are presented as mean ± standard deviation, median interquartile (25th–75th) for the continuous data, or frequency (percents) for the categorical data.

Abbreviations: %HRmaxpred, percentage of predicted maximum heart rate at the peak of exercise; EIB, exercise‐induced bronchoconstriction; FEV1, forced expiratory volume in 1 s; FVC, forced vital capacity; HRp, heart rate at peak of exercise; SpO2, partial saturation in oxygen.

^a^ *p*‐value of Student *t*‐test.

^b^ *p*‐value of *U* Mann–Whitney test.

^c^ *p*‐value of Chi‐square test.

^d^ *p*‐value of Fisher test with Bonferroni correction.

### 3.3 Cardiovascular and respiratory responses to the ISWRT and EIB prevalence

**FIGURE 3:**
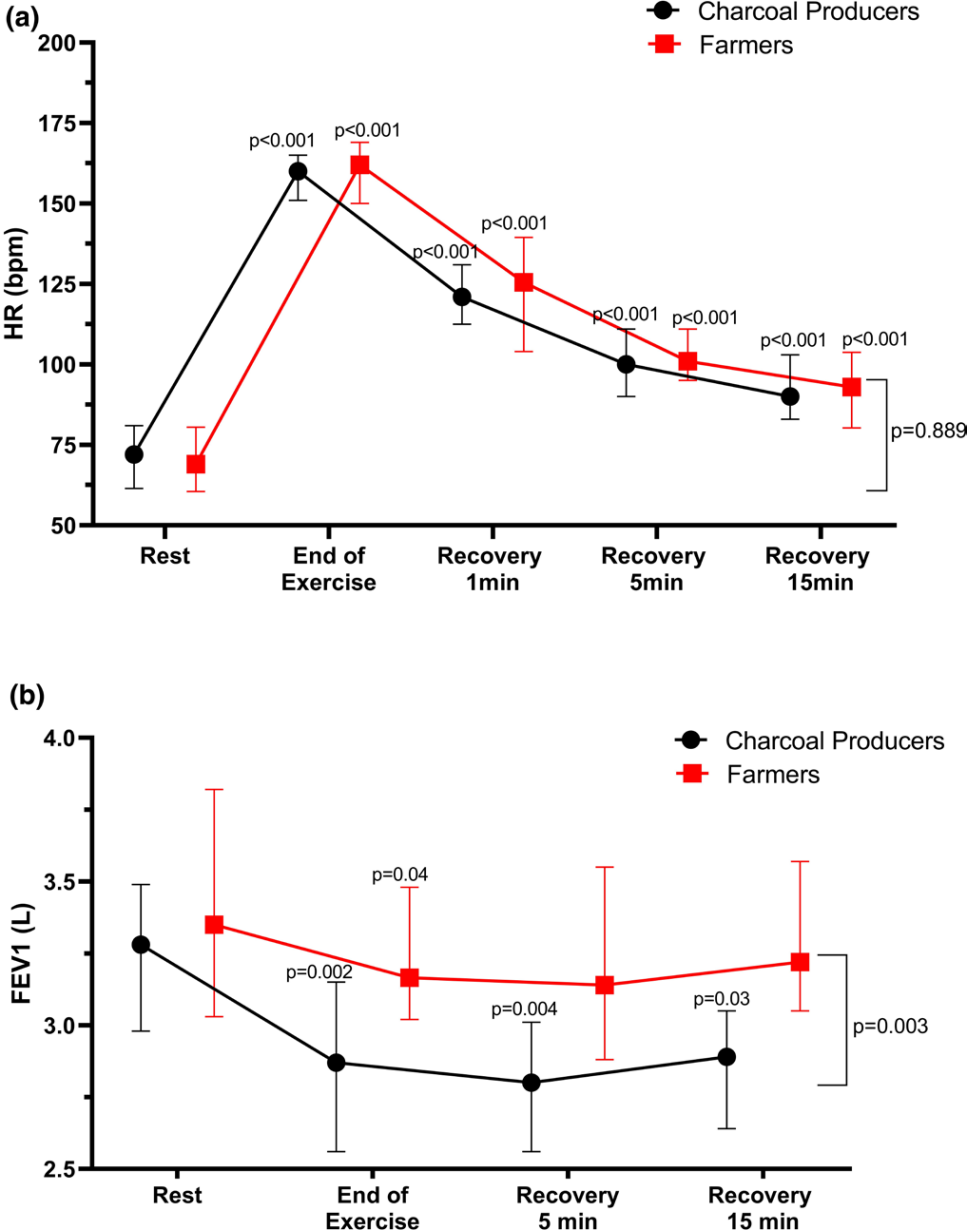
Cardiopulmonary response at the end of exercise test and during recovery. (a) HR: Heart rate. (b) FEV1: Forced expiratory volume in 1 s. Data are presented as medians interquartile range. The comparisons were performed using Friedman's test with Dunn's correction for HR, or ANOVA mixed model with Bonferroni correction for FEV1. *p*‐value above each time represents time effect within each group in comparison to resting value, while next to bars represents the group effect.

CPs and farmers groups exhibited similar HR and blood pressure (SBP and DBP) responses to the ISWRT (Tables 2 and 3). Additionally, HR did not return to its resting value in both groups even 15 min post‐exercise (*p* < 0.001; Figure 3A).

**TABLE 3:** Comparisons of blood pressure between charcoal producers and farmers at rest, at the end of exercise, and during recovery.

	HR, bpm		*p*‐value	SBP, mmHg		*p*‐value	DBP, mmHg		*p*‐value
	CP (*n* = 45)	Farmers (*n* = 36)		CP (*n* = 45)	Farmers (*n* = 36)		CP (*n* = 45)	Farmers (*n* = 36)	
Rest	72 (62–81)	69 (61–78)	0.51	119 (110–135)	118 (110–127)	0.70	70 (70–85)	76 (70–80)	0.83
End of exercise	160 (152–165)	162 (150–169)	0.73	166 (159–180)	166 (159–175)	0.89	70 (67–80)	75 (70–81)	0.56
1 min recovery	120 (110–131)	126 (106–139)	0.52	145 (130–160)	147 (139–157)	0.94	75 (70–80)	78 (74–86)	0.26
5 min recovery	100 (90–111)	101 (95–111)	0.89	125 (117–130)	125 (120–129)	0.86	71 (68–79)	75 (72–80)	0.10
15 min recovery	90 (84–103)	93 (80–104)	0.64	116 (110–123)	120 (115–124)	0.20	70 (69–77)	73 (70–80)	0.08

*Note*: To harmonize presentation, data are presented as median, interquartile interval (25th–75th).

Abbreviations: CP, charcoal producers; DBP, diastolic blood pressure; HR, heart rate; SBP, systolic blood pressure.

At the end of exercise, the FEV1/FVC ratio decreased compared to baseline in CPs (79 ± 12 vs. 74.2 ± 17.2, p < 0.001) but not in Farmers (81 ± 10 vs. 81.4 ± 20.6, *p* = 0.834). While FVC remained unchanged in both groups (CPs: 3.9 (3.5–4.7) vs. 3.9 (3.3–4.4), *p* = 0.257); Farmers: (4.2 (3.8–4.8) vs. 4.1 (3.5–4.9), *p* = 0.875). Both the CPs and farmers showed lower FEV1 immediately after exercise compared to resting values (*p* < 0.05) (Table 2). However, FEV1 remained lower in CPs than in farmers during the recovery period (*p* < 0.01; Figure 3B). In CPs, the mean change (% change) in FEV1 was −0.3 ± 0.6 L (−10.6%) at the end of ISWRT, −0.3 ± 0.6 L (−10.0%) after 5 min of recovery, and − 0.3 ± 0.7 L (−8.1%) after 15 min of recovery. In Farmers, the corresponding values were − 0.2 ± 0.5 L (−6.3%) at the end of ISWRT, −0.2 ± 0.5 L (−5.1%) after 5 min of recovery, and − 0.1 ± 0.4 L (−3.7%) after 15 min of recovery. The Farmer group recovered their resting FEV1 value after 5 min of recovery, whereas the CPs group still presented a reduced FEV1 even after 15 min of recovery (*p* < 0.05; Figure 3B). The prevalence of EIB was higher in CPs (60%) compared to farmers (27.8%, *p* = 0.006; Table 2).

## 4 DISCUSSION

Our results indicate that the AQI is poor in charcoal workplaces, and CPs are more intoxicated with biomass pollutants but demonstrate comparable aerobic capacity and cardiovascular adaptations to exercise than farmers. However, they exhibit a more significant and prolonged decrease in FEV1 following the ISWRT, as well as a higher prevalence of EIB, compared to farmers.

At baseline, CPs and farmers were comparable in terms of sociodemographic and anthropometric characteristics, with normal and comparable cardiovascular and spirometry measurements. Both groups were relatively young, healthy, with normal weight and engaged in high levels of weekly PA. These similarities, combined with previously documented high PA at work and walking in African rural peoples (Guthold et al., 2011), likely explain the comparable distance covered during the ISWRT by both groups. Furthermore, both groups reported similar levels of dyspnea, and the reasons for stopping exercise were not different, suggesting no difference in exercise tolerance.

Although CPs exhibited high CO intoxication due to charcoal biomass exposure at their workplaces, they covered the same distance as Farmers, which was higher than reported for the 15‐meter ISWRT in healthy young adults not exposed to pollution (Mikawa & Senjyu, 2011; Probst et al., 2012). This suggests satisfactory implications for our CPs and farmers who exhibited estimated VO2 max within the expected range for their age and gender (Léger & Lambert, 1982; Mikawa & Senjyu, 2011), and perhaps as all were chronically exposed to occupational pollution, even if they had different sources. It is, however, known that even short‐term exposure to polluted air may negatively affect exercise capacity (Kargarfard et al., 2015; You et al., 2022). Interestingly, the high and extended exposure of CPs to charcoal biomass pollutants did not seem to impact their exercise capacity. This observation might suggest that the benefits associated with high weekly PA offset the potential adverse effects of pollution. Another explanation will be that these subjects chronically exposed to occupational charcoal pollution (about 5 years) might, in the long term, become less sensitive to the biomass pollutants' negative effects on exercise capacity. Indeed, Tainio et al. mentioned that exercising regularly in a polluted environment may not have negative effects on physical performance in trained subjects (Tainio et al., 2016).

Blood pressure returned nearly completely to resting values after 5 min of recovery, which might be considered normal after exercise (Staes et al., 2024). HR recovery (HRR) after maximal exercise testing is usually observed within the first minutes after exercise, and insufficient HRR has been related to cardiovascular risk (Jouven et al., 2005; Qiu et al., 2017). Based on this, we can consider that our subject's HRR is normal as they recovered approximately 40 bpm within the first minute of exercise cessation. However, even if the time needed to completely recover resting HR is inconsistently reported in the literature (Buchheit et al., 2007; Peçanha et al., 2014), HR was still above resting value 15 min after ISWRT in both groups, which could be considered as being above a normal range. This observation might indeed be relevant in consideration of a possible impaired restoration of autonomic regulation of the HR (Gold et al., 2000; Lauer, 2009). Chronic exposure to pollution has been shown indeed to affect the slow phase of HRR (Pouriamehr et al., 2023). We question whether incomplete HRR a quarter of an hour after exercise testing could be an early indicator of potential cardiovascular changes in subjects who are chronically exposed to charcoal biomass, as reported in our previous study (Lofuta et al., 2025). This concern would be aligned with findings already reported in healthy subjects with long‐term exposure to pollution from other sources (Pouriamehr et al., 2023; Unosson et al., 2013).

Although FEV1 typically slightly increases after exercise (Staes et al., 2024), we observed a decrease in FEV1 following the completion of the ISWRT in both CPs and farmers. Given our testing was performed under recommended conditions (Hallstrand et al., 2018; Holland et al., 2014; Parsons et al., 2013) and away from any sources of air pollution, this immediate reduction in FEV1 in both groups could be linked to an airway reactivity related to their chronic occupational exposure in their distinctive workplaces (Lofuta Olenga Vuvu et al., 2024).

Farmers are indeed also exposed to fine particles, which could account for the 27.8% prevalence of EIB exceeding the prevalence in the general population (Weiler et al., 2016). This EIB prevalence is, however, still lower than the prevalence of CPs, probably because of the lower level of pollution and from a different source. This lighter pollution might have caused a lighter form of airway irritability. Moreover, farmers' FEV1 recovered to baseline within 5 min after exercise cessation, whereas CPs did not regain their baseline values even after 15 min of recovery, which is a classical sign of persistent EIB (Price, Walsted, et al., 2022; Weiler et al., 2016). Exposure of CPs to extremely high charcoal biomass pollutants during working most probably dramatically exacerbated airway irritability, engaging this persistent EIB.

Moreover, the prevalence of EIB in CPs was twice that in Farmers. Specifically, the 60% prevalence of EIB in CPs surpasses the 15% prevalence typically reported in healthy adults (Reinhard‐Groebli & Nicod, 2017; Weiler et al., 2016) and the 40% prevalence noted in African athletes (Agodokpessi et al., 2012). Furthermore, this rate exceeds the 40% prevalence of EIB reported in athletes usually trained in polluted environments (Levai et al., 2016; Price, Sewry, et al., 2022).

Other studies already indicated that EIB is more pronounced in individuals engaged in intense PA and exposed to pollutants (Couto et al., 2018; Price, Sewry, et al., 2022). Indeed, in our population of CPs, the intense work rate during charcoal production requires hyperventilation, which increases the inhalation of biomass pollutants (smoke and dust), as demonstrated by their high level of EACO, underlining a strong intoxication to smoke from the combustion of wood. This likely exacerbates airway inflammation and reactivity, potentially predisposing individuals to EIB (Swiston et al., 2008).

This study is one of the first evaluations of cardiopulmonary responses to exercise and EIB in subjects performing PA in chronic exposure to charcoal biomass pollutants condition. It offers moreover crucial physiological insights into vulnerable rural healthy subjects, allowing the monitoring of cardiopulmonary health risks in a low‐income country. Nevertheless, this study has certain limitations: while we captured workplace AQI and the participants CO intoxication level, the seniority of exposure was self‐reported and not methodologically controlled, raising the possibility of memory bias in the participants. Furthermore, post‐ISWRT FEV1 measurements used to define EIB were taken within 15 min of recovery, whereas a recovery period of up to 30 min could have contributed to assessing long‐term lung function recovery (Hallstrand et al., 2018; Parsons et al., 2013). Finally, the study could be further strengthened in laboratory exercise testing, such as CPET; this would allow the highlighting of additional data not captured in the current protocol.

## 5 CONCLUSION

Cardiovascular responses and exercise capacity during and after the ISWRT were not significantly different in CPs compared to farmers. However, the slower FEV1 recovery and higher prevalence of EIB observed in CPs suggest that chronic exposure to charcoal biomass pollutants increases airway reactivity to exercise. These findings underscore the impairment of pulmonary function requiring the implementation of policies to monitor exposure to biomass pollutants.

## AUTHOR CONTRIBUTIONS

PLOV, AKM, MK, and GD designed the protocol and acquired the research funding. PLOV implemented the study and collected the data. PLOV, AVM, NP, MK, and GD validated and analyzed the data. PLOV, MK, PVDB, SP‐B, and GD wrote the first manuscript. All authors revised and validated the final version of the manuscript.

## FUNDING INFORMATION

This research was supported by Academie de la Recherche et de l'Enseignement Supérieur (ARES) Belgium grant. The funders were not implicated in the scientific process of this research.

## CONFLICT OF INTEREST STATEMENT

The authors declare no conflict of interest likely to affect the scientific integrity of this article.

## Data Availability

Study dataset is available on request from the corresponding author or to the Laboratory of Rehabilitation Sciences of the Faculty of Human Movement Sciences, ULB.
